# A High‐Performance Garnet‐Based All‐Solid‐State Battery Fabricated Through Room‐Temperature Ultrasonic Welding

**DOI:** 10.1002/advs.202504388

**Published:** 2025-06-24

**Authors:** Tianlu Pang, Shufen Wu, Han Wu, Xiaobao Li, Yande Li, LiTao Yu, Hui Zhang, Yong Han, Zhi Guo, Nian Zhang

**Affiliations:** ^1^ Shanghai Synchrotron Radiation Facility Shanghai Advanced Research Institute Chinese Academy of Sciences Shanghai 201204 China; ^2^ State Key Laboratory of Functional Materials for Informatics Shanghai Institute of Microsystem and Information Technology Chinese Academy of Sciences Shanghai 200050 China; ^3^ Eastern Institute for Advanced Study, Ningbo Key Laboratory of All‐Solid‐State Battery, Zhejiang Key Laboratory of All‐Solid‐State Battery Eastern Institute of Technology Ningbo Zhejiang 315200 China; ^4^ Center for Transformative Science ShanghaiTech University Shanghai 201210 China

**Keywords:** garnet electrolyte, high critical current density, interface, LiMg alloy, ultrasound solid welding

## Abstract

Garnet‐type Li_6.5_La_3_Zr_1.5_Ta_0.5_O_12_ (LLZO) has emerged as a highly promising solid electrolyte for next‐generation Li metal batteries due to its high Li^+^ conductivity and stability against metallic lithium. However, its practical application is hindered by poor interfacial contact between Li and LLZO, as well as the persistent issue of lithium dendrite formation during cycling. In this study, a novel and efficient strategy is proposed to address these challenges by employing a room‐temperature ultrasonic treatment combined with a LiMg alloy anode. The fabricated symmetrical UW‐LiMg/LLZO/UW‐LiMg cell exhibits a low interfacial resistance and achieves an unprecedented critical current density of 4.45 mA cm^−2^. Furthermore, these cells demonstrate excellent cycling stability, maintaining stable lithium plating/stripping for over 1000 h at a high current density of 1 mA cm^−^^2^ with a low overpotential of ≈30 mV. The superior performance is attributed to the enhanced anode ductility achieved through Mg alloying and the formation of an ultra‐stable interface layer. The all‐solid‐state UW‐LiMg/LLZO/LiFePO_4_ battery, incorporating an ultrasonically treated alloy anode and a fluorinated cathode interface, delivers a specific capacity of 153 mAh g^−1^ at 0.5 C and retains an impressive capacity retention of 90% after 200 cycles at room temperature.

## Graphical abstract

A room‐temperature ultrasonic treatment enhances the LiMg/LLZO interface, where the Mg framework and in situ formed Li_2_O/MgO interphase enable a record 4.45 mA cm^−2^ CCD and a stable cycling for over 1000 h at 1 mA cm^−2^. Combined with a fluorinated cathode interface, the ASSB delivers 153 mAh g^−1^ at 0.5 C with 90% capacity retention after 200 cycles.

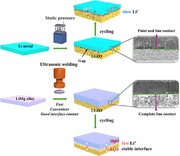

## 1 Introduction

Lithium‐ion batteries (LIBs) have dominated the energy storage landscape for decades, revolutionizing electrochemical energy storage technology. However, their widespread adoption has been hampered by two fundamental limitations: the insufficient energy density and safety concerns associated with flammable liquid organic electrolytes.^[^ ^1^, ^2^ ^]^ These challenges have spurred intensive research into alternative technologies, with all‐solid‐state batteries (ASSBs) emerging as the most promising next‐generation solution.^[^ ^3^, ^4^ ^]^ Among various types of solid‐state electrolytes, garnet‐type electrolyte LLZO has received much attention due to their high ionic conductivity (10^−4^‐10^−3^ S cm^−1^) and wide electro‐chemical window, especially excellent compatibility with Li metal anode.^[^ ^5^, ^6^ ^]^ However, unlike highly flexible solid electrolytes such as argyrodite^[^ ^7^, ^8^, ^9^ ^]^ and complex hydrides,^[^ ^10^ ^]^ which can form well‐bonded interfaces with Li metal anode. LLZO suffers from intrinsic rigidity, resulting in poor interfacial contact with Li metal and high interfacial resistance, while the non‐uniform plating and stripping of Li contribute to the formation of dendrites.^[^ ^11^, ^12^, ^13^, ^14^ ^]^

To address the interfacial challenges in garnet‐based ASSBs, researchers have developed several innovative strategies to enhance the compatibility between the electrolyte and the electrode materials, thereby improving the overall performance and safety of the batteries. These strategies include incorporating advanced additives into the electrolyte,^[^ ^15^, ^16^ ^]^ constructing novel artificial solid electrolyte interphase (SEI) layers,^[^ ^17^, ^18^ ^]^ and developing innovative composite anodes.^[^ ^19^ ^]^ Among these approaches, the utilization of LiMg alloy as an anode material has emerged as particularly promising, owing to its unique combination of properties: high energy density, extensive solid solubility, and enhanced mechanical characteristics.^[^ ^20^, ^21^ ^]^ Compared to conventional anodes, LiMg alloys demonstrate superior electrochemical performance, including more efficient lithium‐ion stripping/plating kinetics, formation of denser interfacial layers, higher critical current density, improved ion diffusion pathways, and effective mitigation of lithium dendrite growth.^[^ ^22^, ^23^, ^24^ ^]^ The alloy's excellent performance can be attributed to its unique phase characteristics, that the body‐centered cubic (BCC) phase can accommodate up to 30% Li across a wide compositional range.^[^ ^25^, ^26^ ^]^ This remarkable phase stability suggests minimal bulk phase transformations during electrochemical cycling, thereby ensuring superior microstructural integrity.^[^ ^27^ ^]^ Furthermore, the LiMg alloy's elevated melting point contributes to enhanced battery safety and improved electrolyte wettability.^[^ ^28^, ^29^ ^]^ These characteristics are crucial for optimizing the performance of ASSBs, yet comprehensive studies remain surprisingly limited for garnet‐based electrolytes in the literature.

Another serious problem is how to achieve good contact between the alloy anode and the garnet‐based electrolyte. Traditional methods such as static pressing and fusion welding have been employed to enhance the interfacial contact, but these approaches often involve complex procedures that hinder practical implementation.^[^ ^30^ ^]^ Other strategies focus on surface modification of ceramic electrolytes. For instance, Han and colleagues demonstrated the effectiveness of atomic layer deposition (ALD) in coating LLZO with an Al_2_O_3_ thin film, which not only improves lithium wettability but also establishes mixed ion‐electron conduction pathways.^[^ ^31^, ^32^ ^]^ However, the techniques face scalability challenges for mass production due to their high cost and time‐consuming processes. The application of ultrasonic welding has proven to be an effective and innovative method for enhancing the interfacial contact between metal and oxide ceramic.^[^ ^33^, ^34^, ^35^, ^36^, ^37^ ^]^ This innovative technique utilizes high‐frequency acoustic vibrations that transform into frictional and deformation energies at the interface, accompanied by localized temperature increases. These synergistic effects promote the formation of robust interfacial bonds, particularly at metal‐ceramic interfaces, thereby demonstrating significant potential for optimizing the Li/garnet interface.^[^ ^35^, ^38^, ^39^, ^40^ ^]^

In this study, we employed a straightforward room‐temperature ultrasonic‐assisted welding method to modify the interface of LiMg alloy anodes and LLZO ceramic pellets. The assembled battery showcased excellent electrochemical performance, achieving a notable critical current density of 4.45 mA cm^−^^2^. Remarkably, the Li‐Li symmetric battery maintained stable cycling for over 1000 h at a high current density of 1 mA cm^−^^2^. Furthermore, the UW‐LiMg/LLZO/LiFePO_4_ full‐cell configuration exhibited consistent performance, enduring 200 cycles at 0.5 C with a discharge capacity of 153 mAh g^−1^ and retaining a capacity retention of 90% at room temperature.

## 2 Results and Discussion

**Figure 1:**
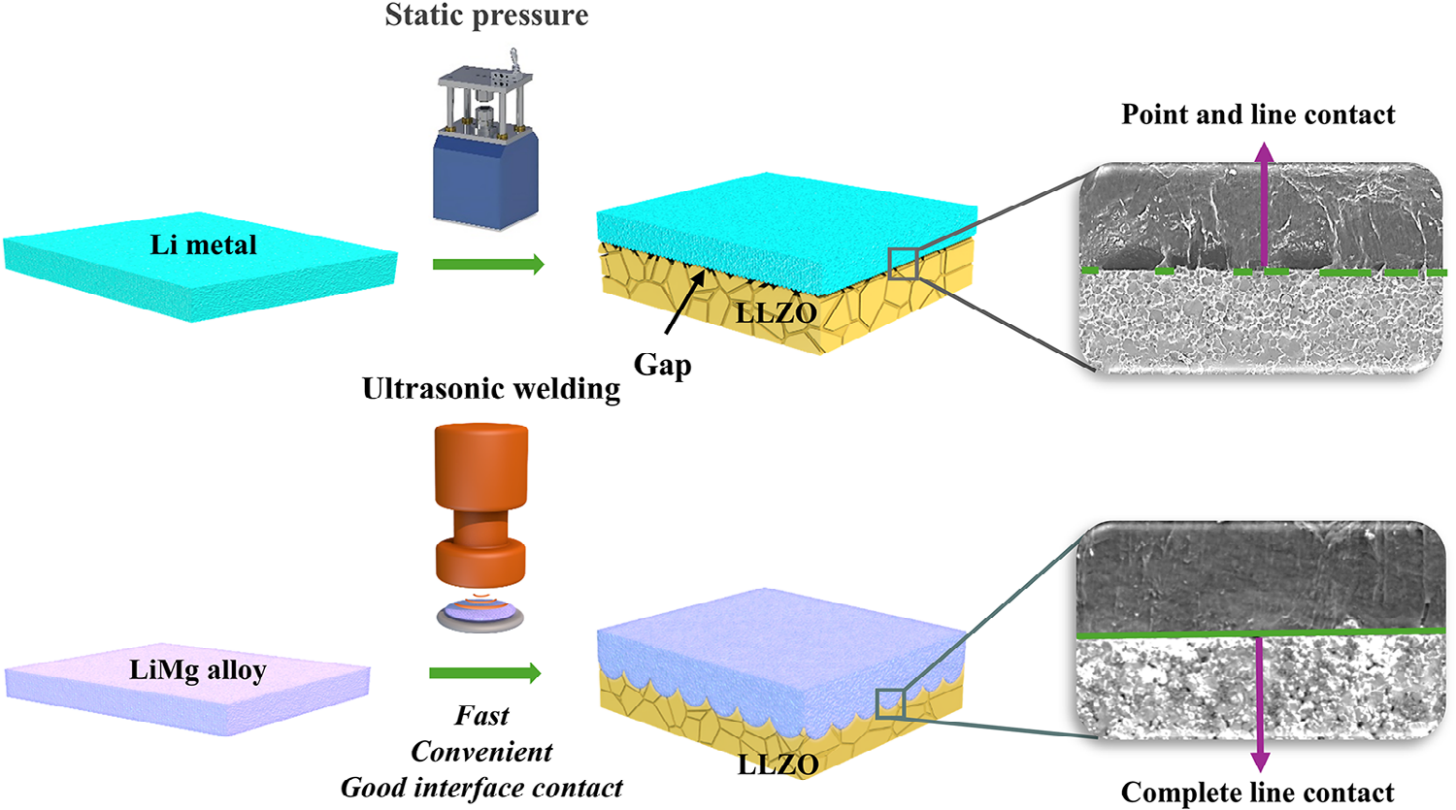
Schematic illustration of the conventional interface construction method alongside our refined interface strategy. The SEM images on the right depict the interfacial morphology of Li/LLZO and UW‐LiMg/LLZO.

The inherent lithiophobic nature of the LLZO surface, particularly when surface contaminants are present, leads to poor Li/LLZO contact and significantly elevated interfacial impedance. Furthermore, the morphological instability and limited ductility of Li metal anodes pose additional challenges to the construction of stable interfaces, ultimately compromising the cycling performance of the ASSBs. As illustrated in **Figure** **1**, the conventional static pressing method, which involves compressing Li metal and LLZO together (denoted as Li/LLZO), often results in significant interfacial voids. Moreover, during the cycling process, stripping 1 mAh cm^−2^ of Li creates a gap of ≈4.9 µm based on the theoretical volumetric capacity of Li metal. These gaps, caused by the poor ductility and sluggish vacancy diffusion kinetics within the Li metal, lead to high interfacial resistance and poor cycling performance.^[^ ^27^ ^]^

To overcome these challenges, a straightforward and effective room‐temperature ultrasonic‐assisted welding technique was utilized to create an optimal contact interface between the anode and the LLZO pellet. As illustrated in Figure S1 (Supporting Information), LiMg alloy films containing 20 wt% Mg were utilized instead of pure Li metal to enhance both the structural stability of the anode and the interfacial kinetics. Within an argon‐filled glove box, LiMg alloy films were layered onto the LLZO surface. A portable ultrasonic device, as depicted in Figure S1 (Supporting Information), was then applied to the anode membrane on the LLZO pellet for 30 s, significantly improving the interfacial contact. The resulting sample was designated as UW‐LiMg/LLZO. Cross‐sectional scanning electron microscope (SEM) images, displayed in the right panel, reveal the interfacial morphology of Li/LLZO and UW‐LiMg/LLZO. The Li/LLZO interface exhibits substantial voids, a consequence of the inherent hardness of pure Li metal. In contrast, the UW‐LiMg/LLZO interface shows significantly fewer voids, demonstrating that the enhanced mechanical properties of the LiMg alloy contribute to superior interfacial bonding. This comparison clearly indicates that the combination of LiMg alloy and the ultrasonic welding technique substantially improves the integrity of the welded interface.

**Figure 2:**
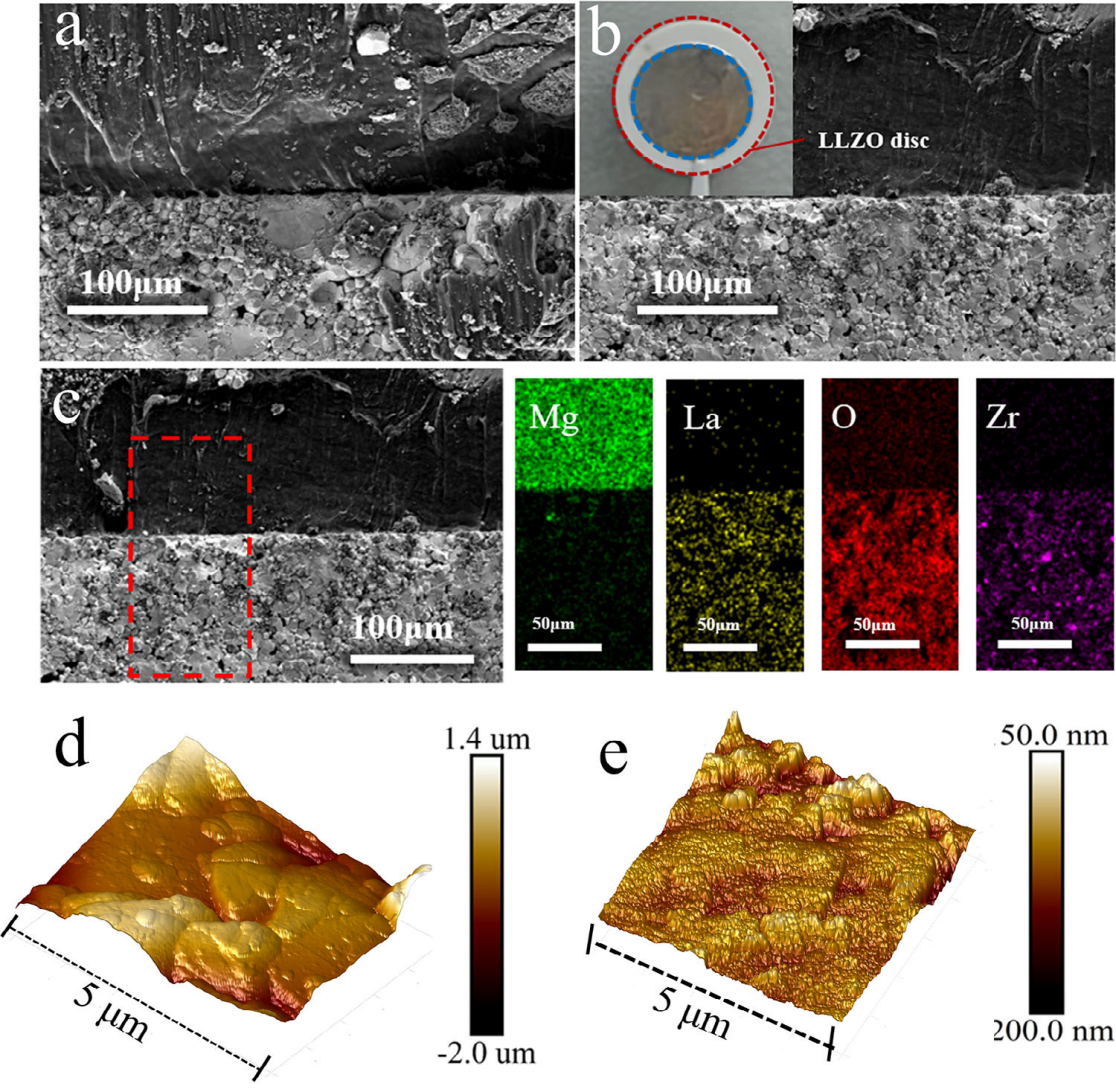
a) Cross‐sectional SEM image of the LiMg/LLZO interface fabricated using the conventional static pressing method. b) Cross‐sectional SEM image of the UW‐LiMg/LLZO interface. c) Enlarged cross‐section SEM image and EDS elemental mappings for UW‐LiMg/LLZO. d) AFM image of pristine LLZO surface. e) AFM image of the UW‐LiMg/LLZO surface.

To provide a more detailed comparison of the interfacial differences resulting from ultrasonic treatment and static pressing, we further analyzed the interfacial morphology and elemental distribution using cross‐sectional SEM, energy‐dispersive X‐ray spectroscopy (EDS) and atomic force microscope (AFM). The UW‐LiMg/LLZO interface in **Figure** **2**b shows intimate contact with no microscopic voids, signifying superior interfacial bonding. In contrast, the LiMg/LLZO interface formed by static pressing in Figure 2a still displays noticeable gaps. Additionally, as shown in Figure 2c, post‐ultrasonic treatment, the LiMg alloy is smooth and firmly attached to the LLZO pellet surface, affirming that the room‐temperature ultrasonic‐assisted welding technique can significantly enhance the interfacial contact between metals and ceramics. The EDS elemental mappings of the selected cross‐sectional area in Figure 2c, along with the EDS elemental line scan profile in Figure S3 (Supporting Information), reveal a well‐defined and distinct UW‐LiMg/LLZO interface. It is observed that the elements Zr, O, and La, corresponding to the LLZO pellet, exhibit a consistent distribution, while Mg demonstrates slight diffusion from the LiMg anode into the surface thin layer of the LLZO.

The AFM images in Figure 2d and the SEM images in Figure S2 (Supporting Information) reveal that the original LLZO particles are relatively large and their surfaces are partially covered with Li_2_CO_3_,^[^ ^41^ ^]^ resulting in poor surface planarity with roughness values ranging between 2 and 3 µm. Ultrasonic treatment achieves two critical improvements: first, it fragments these large particles, reducing surface roughness to 50–100 nm (shown in Figure 2e); second, it enables the compliant LiMg alloy anode to infiltrate interparticle gaps created by the fragmentation process. This synergistic effect facilitates the formation of a stable, densely integrated interfacial layer with excellent performance.

**Figure 3:**
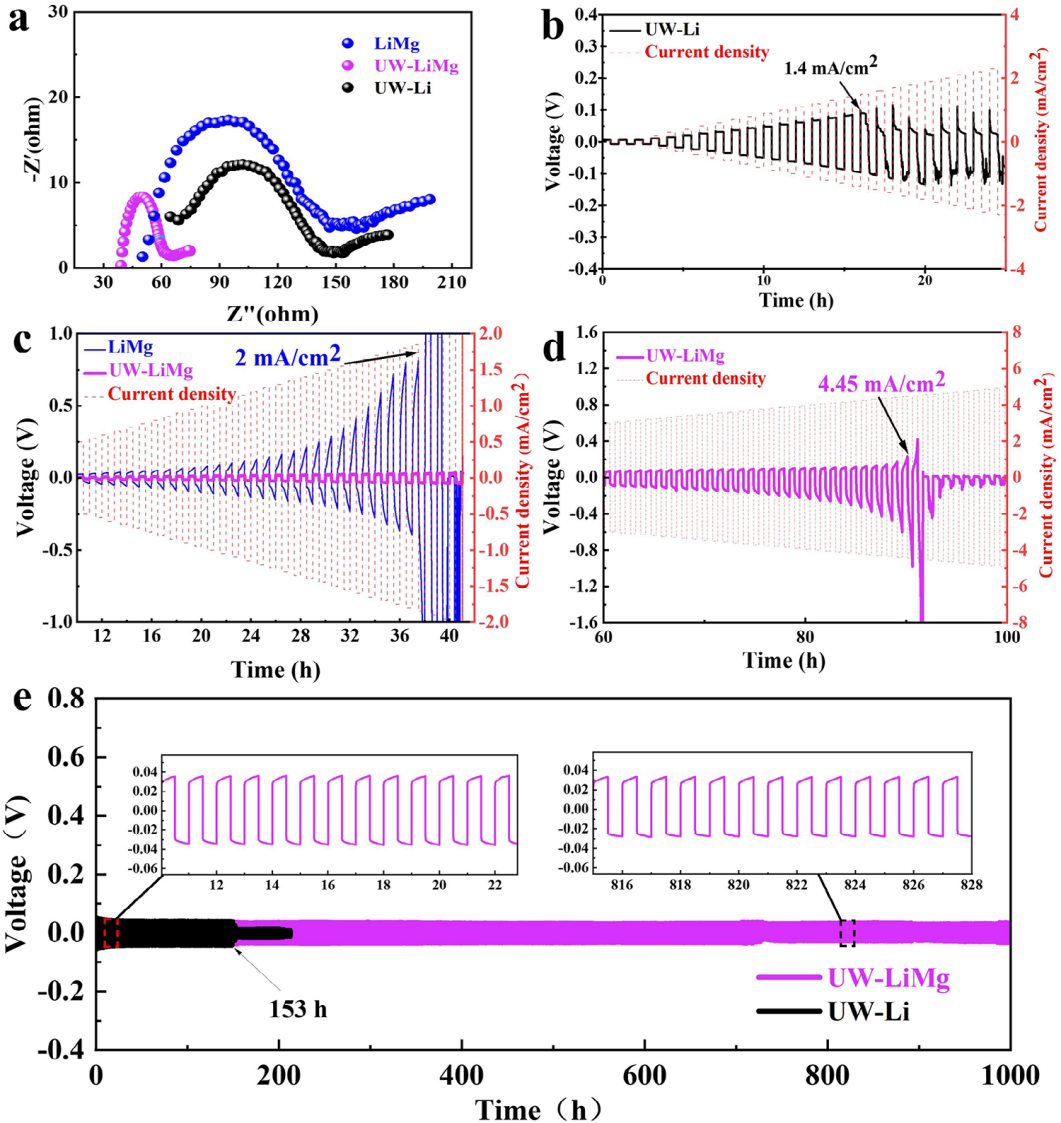
a) Electrochemical impedance spectra (EIS) of symmetric cells assembled with LiMg, UW‐Li, and UW‐LiMg anodes. b) CCD test results of UW‐Li/LLZO/UW‐Li, c) LiMg/LLZO/LiMg, UW‐LiMg/LLZO/UW‐LiMg and d) UW‐LiMg/LLZO/UW‐LiMg cells. e) Galvanostatic cycling comparison between UW‐LiMg/LLZO/UW‐LiMg and UW‐Li/LLZO/UW‐Li cells at a high current density of 1 mA cm^−2^.

To assess the influence of ultrasonic treatment on electrochemical performance, symmetric cells of LiMg/LLZO/LiMg, UW‐Li/LLZO/UW‐Li, and UW‐LiMg/LLZO/UW‐LiMg were assembled to evaluate their interfacial resistance, as illustrated in **Figure** **3**a. The ultrasonically treated LiMg alloy anode exhibits significantly lower interfacial impedance ≈65 Ω, compared to both the untreated LiMg alloy and the ultrasonically processed pure Li anode. Furthermore, this value is superior to those reported in previous studies, as comprehensively compared in Table S1 (Supporting Information). This enhancement demonstrates that ultrasonic treatment effectively optimizes the interfacial contact between the LiMg alloy anode and the LLZO electrolyte.

Galvanostatic critical current density (CCD) is a benchmark to evaluate the electroplating and stripping ability of Li at the interface under gradually increasing current density. The current density was systematically increased by a fixed increment after each cycling step, with each step maintained for 1 h at 100 °C. As shown in Figure 3b, the UW‐Li/LLZO/UW‐Li cell exhibits a short circuit at a maximum current density of 1.4 mA cm^−^^2^, representing a significant improvement compared to untreated Li metal ≈0.7 mA cm^−^^2^.^[^ ^18^ ^]^ When Li metal is replaced with a LiMg alloy, even without ultrasonic treatment, the CCD substantially increases to ≈2 mA cm^−2^ as shown in Figure 3c. This enhancement confirms the critical role of Mg incorporation in optimizing the electrode/electrolyte interface. Remarkably, the ultrasonically treated LiMg alloy achieves an ultra‐high CCD of  4.45 mA cm^−2^ (shown in Figure 3d) with a minimal overpotential. The overpotential at the point of short circuit is only ≈0.4 V, highlighting the exceptional interfacial stability and dendrite suppression capability of the ultrasonically treated LiMg alloy. These results emphasize the synergistic advantages of Mg alloying and ultrasonic treatment in advancing the electrochemical performance of solid‐state batteries.

The cycling stability of the assembled cells was assessed through galvanostatic cycling measurements at a relatively high current density of 1 mA cm^−^^2^. The ultrasonically treated Li metal anode exhibits a low cycling overpotential of ≈40 mV. However, a short circuit occurs after only 152 h of operation. This result suggests that, while ultrasonic treatment enhances interfacial contact, the interface remains vulnerable to degradation caused by Li⁺ migration and volumetric changes in the Li metal anode. These factors can lead to an uneven spatial electric field, thereby promoting lithium dendrite growth. In contrast, the UW‐LiMg/LLZO/UW‐LiMg symmetric cell demonstrates excellent stability, maintaining a consistent voltage profile with minimal overvoltage fluctuations for over 1000 h. The nearly identical overpotentials observed at 20 h and 820 h, as illustrated in Figure 3e, confirm the long‐term cycling stability of the interface formed by ultrasonic welding. Furthermore, these findings demonstrate the critical role of Mg in mitigating interface volume changes and suppressing dendrite formation during cycling.

**Figure 4:**
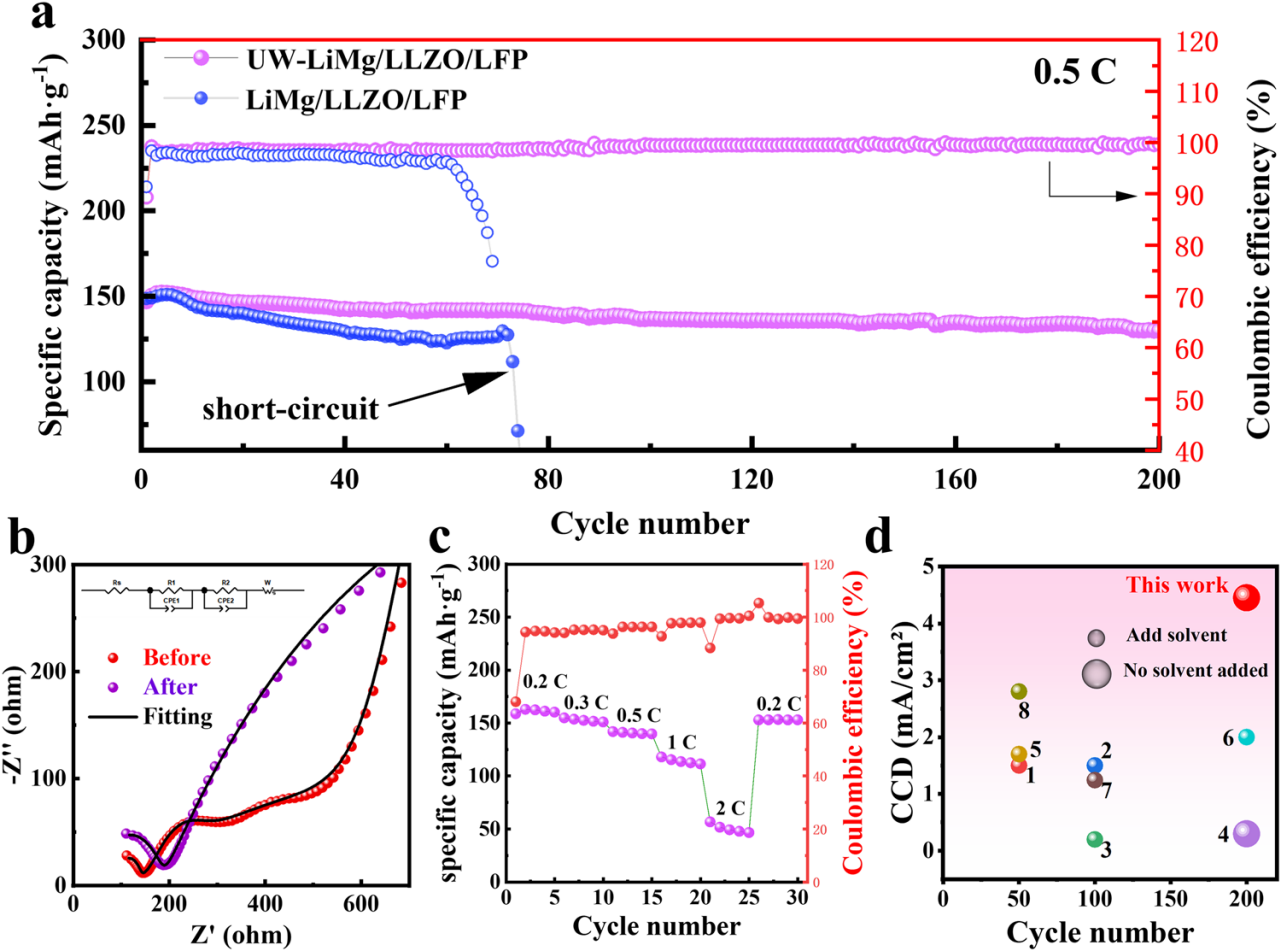
a) Cycling Performance of all‐solid‐state UW‐LiMg/LLZO/LFP and LiMg/LLZO/LFP battery at 0.5 C and room temperature. b) Variation in interfacial impedance of UW‐LiMg/LLZO/LFP cell after 200 cycles. The equivalent circuit fitting results of the EIS spectra are shown in Table S2 (Supporting Information). c) Rate performance of the UW‐LiMg/LLZO/LFP battery at 0.5 C and room temperature. d) Comparison of our electrochemical performance with results reported in other literature (1,^[^ ^42^ ^]^ 2,^[^ ^43^ ^]^ 3,^[^ ^44^ ^]^ 4,^[^ ^45^ ^]^ 5,^[^ ^46^ ^]^ 6,^[^ ^47^ ^]^ 7,^[^ ^48^ ^]^ 8^[^ ^49^ ^]^).

The cycling performance of the all‐solid‐state UW‐LiMg/LLZO/LiFePO_4_ (LFP) battery is presented in **Figure** **4**. To address the poor interfacial contact between LLZO and the LFP cathode, the LLZO surface on the cathode side was treated using a decarbonization‐fluorination strategy prior to battery assembly, as detailed in the experimental section. Figure 4a shows the cycling performance of the assembled UW‐LiMg/LLZO/LFP battery at 0.5 C and room temperature. The initial discharge specific capacity is ≈147 mAh g^−1^, which, after ≈5 activation cycles, reaches a maximum of 153 mAh g^−1^. The ultrasonically treated battery demonstrates exceptional stability, sustaining over 200 cycles with a capacity retention of 90% and a coulombic efficiency exceeding 99.7%. In contrast, untreated samples exhibit rapid capacity degradation, dropping to zero after ≈70 cycles. Figure 4b illustrates the interfacial impedance variation after 200 cycles; the equivalent circuit fitting results of the EIS spectra are shown in Table S2 (Supporting Information). The fitting results demonstrate that the interfacial impedance slightly increases from 43 Ω to 115 Ω after 200 cycles, confirming the successful construction of a robust electrode/electrolyte interface. Moreover, the LiMg alloy effectively suppresses interfacial void formation, thereby stabilizing the interfacial resistance and significantly enhancing the cycling stability of the battery. Figure S4 (Supporting Information) displays cycling performance and the charge‐discharge curves of the UW‐LiMg/LLZO/LFP battery at 0.2 C, further emphasizing the excellent interfacial performance of the full cell. The overpotential shows almost no significant increase after 200 cycles. Figure 4d shows the comparison of our cycling performance with that documented in other literature. In most literature, the addition of liquid electrolyte on the cathode side is required to achieve cycling performance, whereas our system demonstrates optimal cycling performance without the need for any liquid electrolyte. These results highlight the effectiveness of the ultrasonic treatment and interface optimization strategies in achieving long‐term cycling stability and high performance ASSBs.

The rate performance of the battery is illustrated in Figure 4c. The initial discharge specific capacities of UW‐LiMg/LLZO/LFP cells at 0.2 C, 0.3 C, 0.5 C, 1 C and 2 C (1 C = 170 mA g^−1^) are ≈159, 154, 148, 123, and 54 mAh g^−1^, respectively. When the current density is returned to 0.2 C, the discharge specific capacity recovers to ≈156 mAh g^−1^, demonstrating excellent rate performance. This indicates that the introduction of the LiMg alloy through ultrasonic treatment enhances interfacial kinetics and facilitates faster Li⁺ transfer at the interface. These results highlight the outstanding performance of our approach, providing an effective and practical strategy as well as material insights for the future development of solid‐state batteries.

**Figure 5:**
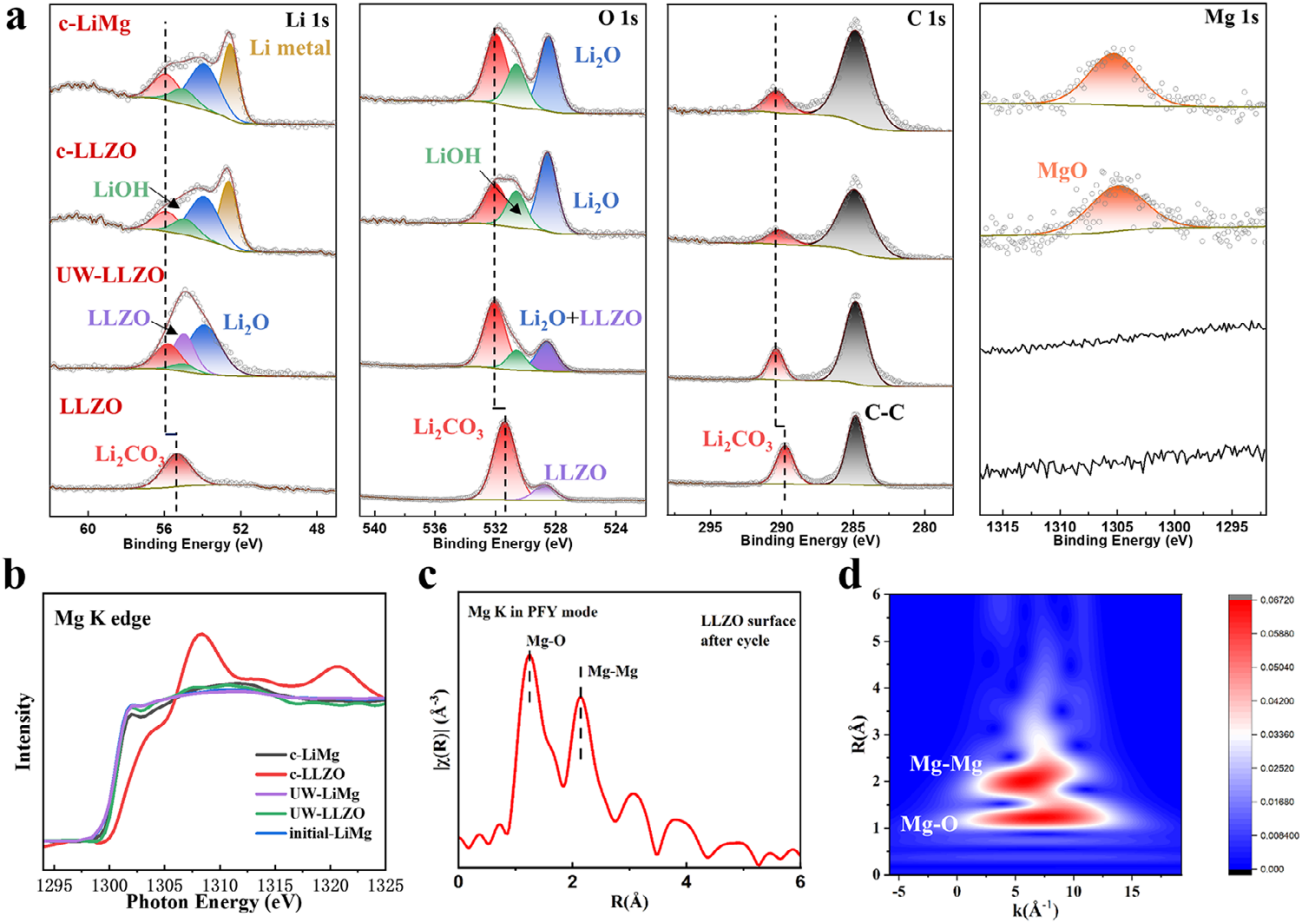
a) Li 1s, O 1s, C 1s, and Mg 1s XPS spectra of pristine LLZO surface, LLZO surface after ultrasonic treatment (UW‐LLZO), UW‐LLZO surface after 50 cycles (c‐LLZO) and UW‐LiMg alloy surface after 50 cycles (c‐LiMg). b) Mg K edge XANES of pristine LiMg alloy, UW‐LLZO, UW‐LiMg, c‐LLZO and c‐LiMg. c) Fourier transform plots derived from the k^3^‐weighted EXAFS spectra at the Mg K‐edge for c‐LLZO. d) Wavelet transform plots of the k^3^‐weighted EXAFS signals for c‐LLZO.

To elucidate the mechanism underlying the ultra‐stable UW‐LiMg/LLZO interface, X‐ray photoelectron spectroscopy (XPS) and X‐ray absorption spectroscopy (XAS) were employed. As shown in **Figure** **5**a, the Li 1s, O 1s, and C 1s XPS spectra reveal that the initial LLZO surface is predominantly covered by a thin Li_2_CO_3_ layer. Notably, after ultrasonic treatment, the characteristic peaks of Li_2_CO_3_ on the LLZO surface shifted ≈0.4 eV toward higher binding energy compared to LLZO and surface contaminant carbon. This phenomenon can be attributed to the following mechanism: Li_2_CO_3_, initially formed by the reaction of the LLZO surface with moisture and CO_2_,^[^ ^50^ ^]^ undergoes partial decomposition and structural modification under ultrasonic treatment. Following ultrasonic treatment, the Li_2_CO_3_ layer is dislodged from the LLZO surface, breaking into nanoparticles and intermixing with other compounds generated through its interaction with the LiMg alloy. This process reduces surface conductivity, inducing charging effects and consequently shifting the binding energy to a higher value. These findings are consistent with our SEM and AFM results, further confirming the critical role of the ultrasonic treatment step in constructing a smooth and dense interfacial layer.

The XPS results in Figure 5a and O K‐edge XAS spectra in total electron yield mode (TEY) with a probing depth ≈10 nm in Figure S5 (Supporting Information) demonstrate that an interfacial layer primarily composed of Li_2_O mixed with residual Li_2_CO_3_ is formed at the interface between LLZO and the LiMg alloy after ultrasonic treatment. The absence of Mg 1s signals throughout the ultrasonic process indicates that Mg is not the primary surface component involved in the interfacial mixing with Li_2_CO_3_ during the ultrasonic process. From the post‐cycling Li 1s and O 1s XPS spectra, it is evident that the compositions on the LLZO side and the LiMg alloy side are highly similar. The distinct signal of Li metal in the Li 1s spectrum^[^ ^51^ ^]^ confirms that metallic lithium can achieve stable plating and stripping at the interfacial layer formed under ultrasonic treatment. These findings highlight the critical role of ultrasonic treatment in creating a stable and functional interface, which facilitates efficient Li^+^ transport and enhances the overall performance of the solid‐state battery.

Mg K edge extended X‐ray absorption fine structure (EXAFS) approach is uniquely technical for probing Mg local coordination environments, providing crucial insights into the dynamic structural changes occurring in alloy‐based anode and their interfaces with electrolytes at different conditions. Due to the weak Mg 1s signals detected by XPS in cycled samples, we utilized Mg K edge XAS in partial fluorescence yield mode (PFY), which offers higher sensitivity and greater probing depth ≈200 nm, to further investigate the role of Mg at the interface. As shown in Figure 5b, the X‐ray absorption near‐edge structure (XANES) spectra of the initial LiMg alloy, UW‐LLZO, UW‐LiMg and c‐LiMg alloy are remarkably similar, indicating that Mg maintains a highly stable structure within the bulk LiMg alloy. However, the Mg K‐edge absorption spectrum of the LLZO surface after cycling exhibits significant changes. Fourier transform and wavelet transform analyses of the k^3^‐weighted EXAFS spectrum, as shown in Figure 5c,d, reveal that Mg on the LLZO surface after cycling exists in a mixed state, partially as metallic Mg and partially as MgO. These findings suggest that Mg undergoes partial oxidation during cycling, forming MgO, while retaining some metallic character at the interface. Recent studies show that MgO introduction at LLZO grain boundaries enhances interfacial mechanical strength by over 60% and increases the CCD by 2–3 times.^[^ ^52^ ^]^ Furthermore, Mg/MgO incorporation at the LLZO/electrode interface has been demonstrated to effectively reduce interfacial resistance and improve cycling stability.^[^ ^53^, ^54^, ^55^ ^]^ Thus, the mixed state of Mg at the LiMg/LLZO interface further enhances interfacial stability, while the preservation of the bulk LiMg alloy's intact structure is crucial for ensuring long‐term cycling stability.

Our findings demonstrate that ultrasonic treatment effectively disrupts the bonding between Li_2_CO_3_ and LLZO while simultaneously integrating these components with the surface material of the LiMg alloy. This process eliminates interfacial voids and constructs a smooth, cohesive interfacial layer. During cycling, a small amount of Mg from the alloy surface diffuses into the interfacial layer, forming metallic Mg and MgO, which further stabilizes the interfacial structure. Concurrently, the Mg within the bulk alloy retains its original state, ensuring the structural integrity of the alloy during lithium plating and stripping processes. Together, these synergistic effects enable the formation of an ultra‐stable UW‐LiMg/LLZO interlayer that results in high‐performance ASSBs.

## 3 Conclusion

In this work, we demonstrate a room‐temperature ultrasonic strategy to address interfacial challenges between metallic anodes and ceramic LLZO electrolytes. By replacing Li metal with a ductile LiMg alloy, interfacial compatibility is significantly enhanced. The ultrasonic process, leveraging high‐frequency oscillations and transient thermal effects, fractures residual Li₂CO₃ layers into nanoparticles on LLZO and facilitates LiMg alloy infiltration into interfacial nanogaps, resulting in a stabilized and low‐roughness interface. This optimized interface enables Li‐Li symmetric cells to achieve exceptional cycling stability, operating for over 1000 h at a high current density of 1 mA cm^−^^2^ with minimal polarization. Furthermore, Li dendrite formation is effectively suppressed, achieving a record critical current density of 4.45 mA cm^−^^2^. XPS and XAS analyses reveal that the Mg framework maintains structural integrity during cycling, mitigating volume fluctuations caused by Li⁺ plating and stripping processes. The ultrasonic‐generated Li_2_O‐rich interphase enhances interfacial Li⁺ transport kinetics, while Mg diffusion from the LiMg alloy surface forms MgO nanodomains within the interlayer, further reinforcing interfacial stability. The all‐solid‐state UW‐LiMg/LLZO/LFP cell delivers a high specific capacity of 153 mAh g^−1^ at 0.5 C and room temperature, with 90% capacity retention after 200 cycles. This work demonstrates ultrasonic processing as a highly efficient interface‐engineering strategy and highlights the transformative potential of LiMg alloys as advanced anodes for ultra‐stable and high‐performance all‐solid‐state batteries.

## 4 Experimental Section

### Material Synthesis and Ultrasonic Treatment

Ta‐substituted LLZO (Li_6.5_La_3_Zr_1.5_Ta_0.5_O_12_) pellets were synthesized through a two‐step solid‐state reaction. Stoichiometric amounts of Li_2_CO_3_ (Alfa Aesar, 99.9%), La_2_O_3_ (Alfa Aesar, 99.9%), ZrO_2_ (Alfa Aesar, 99.5%), and Ta_2_O_5_ (Aladdin, 99.5%) were mixed with 15 mol% excess Li₂CO₃. The mixture was ball‐milled in 2‐propanol for 12 h using agate balls in an agate vial, followed by drying and calcination at 1150 °C for 12 h in the air. The ball‐milling process was repeated, and the resulting powder was sieved through a 600‐mesh screen to obtain fine particles. The LLZO pellets were fabricated by hot‐pressing the powder at 1050 °C under a constant pressure of 50 MPa for 1 h in a flowing argon atmosphere. The synthesized ceramic pellets were promptly transferred and stored in an argon‐filled glove box to prevent exposure to ambient moisture and CO_2_. The LiMg alloy (20 wt% Mg) used in this study was sourced from Tianjin Zhongneng Lithium Industry. Ultrasonic treatment of the anode was performed using a JEKEN CE‐9600 ultrasonic device (70 W, 50 kHz). For the treatment, the anode sheet was placed on a polished LLZO pellet, covered with a Polyethylene Separator (PE) separator, and pressed uniformly using the ultrasonic probe. The ultrasonic welding process was completed within 30 s.

### Material Characterization

The morphological characteristics of the materials were investigated using scanning electron microscopy (SEM, ZEISS Sigma 300) operated at an acceleration voltage of 10 kV. Elemental distribution across the cross‐section was analyzed through energy‐dispersive X‐ray spectroscopy (EDS) element mapping and line scanning. Surface roughness was quantified using an atomic force microscope (AFM, Bruker Dimension Icon). The crystal structures of the electrolyte powder were characterized by X‐ray diffraction (XRD) on a DX‐2700A diffractometer (Cu Kα radiation, *λ* = 1.5406 Å). The O K edge and Mg K edge XAS spectra were carried out at BL02B02 of the Shanghai Synchrotron Radiation Facility (SSRF) in TEY and PFY mode.^[^ ^56^ ^]^ Spectral deconvolution of the XAS data was performed using the Athena software. Surface chemical composition was determined by X‐ray photoelectron spectroscopy (XPS, Thermo Scientific K‐Alpha).

### Electrochemical Characterization

Fabrication of Li‐Li symmetric cells: LLZO ceramic pellets were carefully polished using sandpaper to achieve a smooth and uniform surface. Pre‐cut LiMg alloy electrodes were subsequently ultrasonically welded onto both sides of the polished pellets. The UW‐LiMg/LLZO/UW‐LiMg symmetric cells were then sealed in a 2032‐type coin cell using a crimping machine. Electrochemical impedance spectroscopy measurements of the fabricated UW‐LiMg/LLZO/UW‐LiMg cells were conducted on an EC‐LAB‐SP‐200 electrochemical workstation, with a frequency range spanning from 1 MHz to 100 mHz and a perturbation amplitude of 10 mV. Critical current density tests were performed on a Neware CT‐4008 workstation at 100 °C.

Assemble of the UW‐LiMg/LLZO/LFP all‐solid‐state battery: Prior to assembling the all‐solid‐state battery, the surface of the LLZO on the cathode side was treated using a decarbonization‐fluorination strategy. This process involved dropwise addition of an appropriate amount of 1 M LiPF_6_ in ethylene carbonate:dimethyl carbonate (1:1 v/v) onto the ceramic surface, followed by incubation on a heating stage at 60 °C for 24 h, the thermal treatment completely eliminates the liquid electrolyte components. The cathode slurry was prepared by thoroughly mixing active materials, carbon black, and polytetrafluoroethylene (PVDF) binder in a weight ratio of 80:15:5, with N‐methyl‐2‐pyrrolidone (NMP) added as the solvent. The resulting slurry was uniformly coated onto an aluminum current collector and dried at 120 °C for 8 h in a vacuum oven to fabricate the working electrodes. All battery assemblies were carried out in an argon‐filled glove box (H_2_O and O_2_ levels < 0.1 ppm) using CR2032 coin cell configurations. The cycling performance of the all‐solid‐state batteries was evaluated using a Neware CT‐4008 battery workstation.

### Statistical Analysis

The EIS spectra were analyzed with ZView (Scribner Associates, Inc, Charlottesville, VA, USA). The peak fitting for XPS spectra were conducted with CasaXPS software. The Mg K edge XAS spectra and wavelet transform plots were integrated with the Athena and hamaFortran software (open source).

## Conflict of Interest

The authors declare no conflict of interest.

## Supporting information



Supporting Information

## Data Availability

The data that support the findings of this study are available from the corresponding author upon reasonable request.
