# Supplementary material for: A High‐Performance Garnet‐Based All‐Solid‐State Battery Fabricated Through Room‐Temperature Ultrasonic Welding
Source: Adv Sci (Weinh). 2025 Jun 24;12(36):e04388. doi: 10.1002/advs.202504388 (PMC12463083; doi:10.1002/advs.202504388)
Supplement: Supplementary file 1 — Supporting Information [file ADVS-12-e04388-s001.docx]

Supporting Information

A High-Performance Garnet-based All-Solid-State Battery Fabricated through Room-Temperature Ultrasonic Welding

Tianlu Pang, Shufen Wu, Han Wu, Xiaobao Li, Yande Li, LiTao Yu, Hui Zhang*, Yong Han*, Zhi Guo* and Nian Zhang*

Tianlu Pang, Shufen Wu, Xiaobao Li, Litao Yu, Prof. Hui Zhang, Prof. Zhi Guo and Prof. Nian Zhang

Shanghai Synchrotron Radiation Facility

Shanghai Advanced Research Institute, Chinese Academy of Sciences

Shanghai 201204, China

E-mail: [zhanghui2023@sari.ac.cn](mailto:zhanghui2023@sari.ac.cn), [guoz@sari.ac.cn](mailto:guoz@sari.ac.cn), [zhangnian@sari.ac.cn](mailto:zhangnian@sari.ac.cn).

Tianlu Pang, Yande Li

State Key Laboratory of Functional Materials for Informatics

Shanghai Institute of Microsystem and Information Technology, Chinese Academy of Sciences

Shanghai, 200050 China

Han Wu

Eastern Institute for Advanced Study

Eastern Institute of Technology Ningbo

Zhejiang 315200, China

Prof. Yong Han

Center for Transformative Science

Shanghai Tech University

Shanghai, 201210, China

E-mail: [hanyong@shanghaitech.edu.cn](mailto:hanyong@shanghaitech.edu.cn)

^#^ Tianlu Pang, Shufen Wu contributed equally.

Keywords: LiMg alloy, Garnet electrolyte, Interface, Ultrasound solid welding, High critical current density.


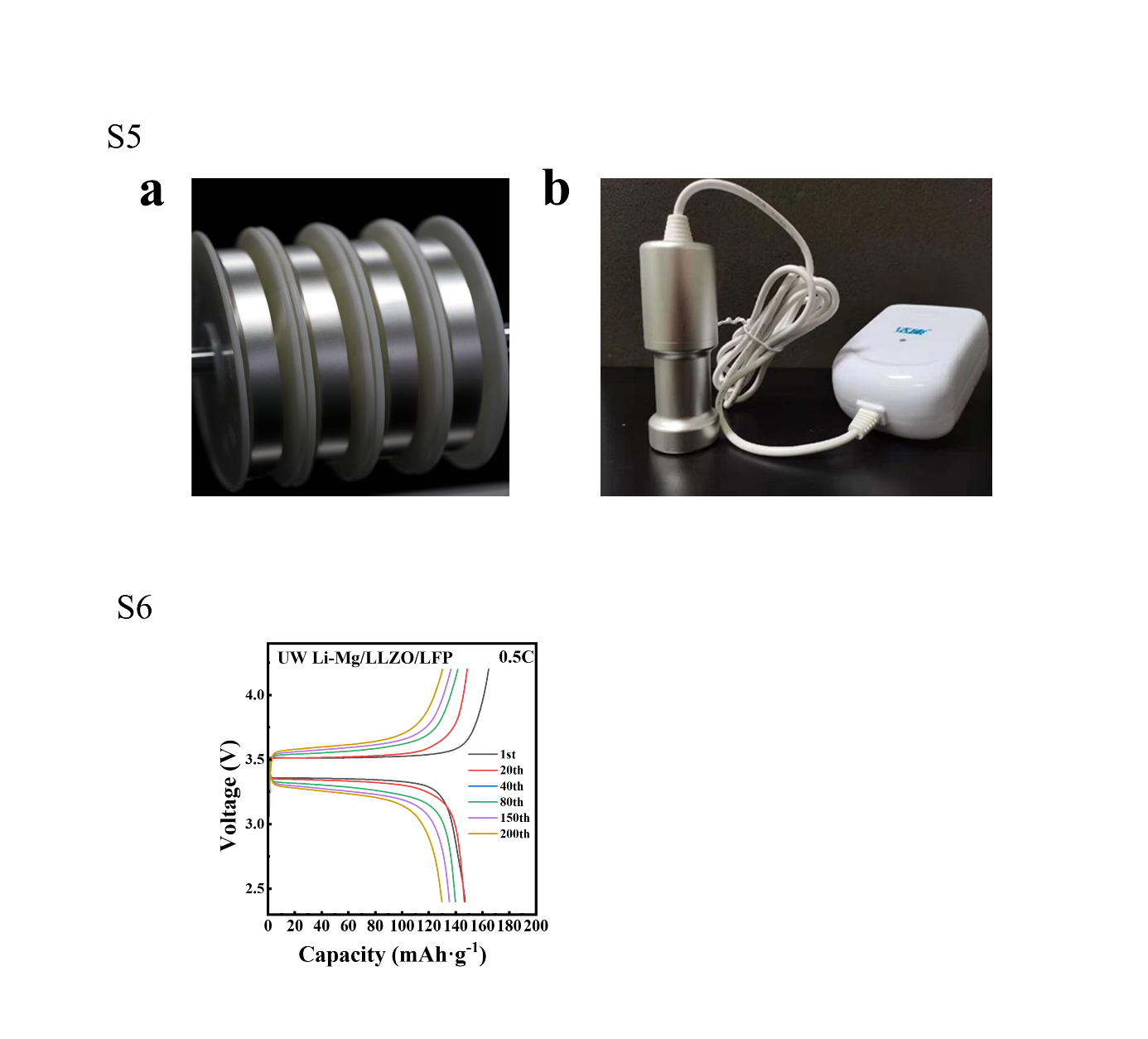


**Figure S1.** Photographs of the a) LiMg alloy strip with 20 wt% Mg and b) ultrasonic equipment utilized in this study.


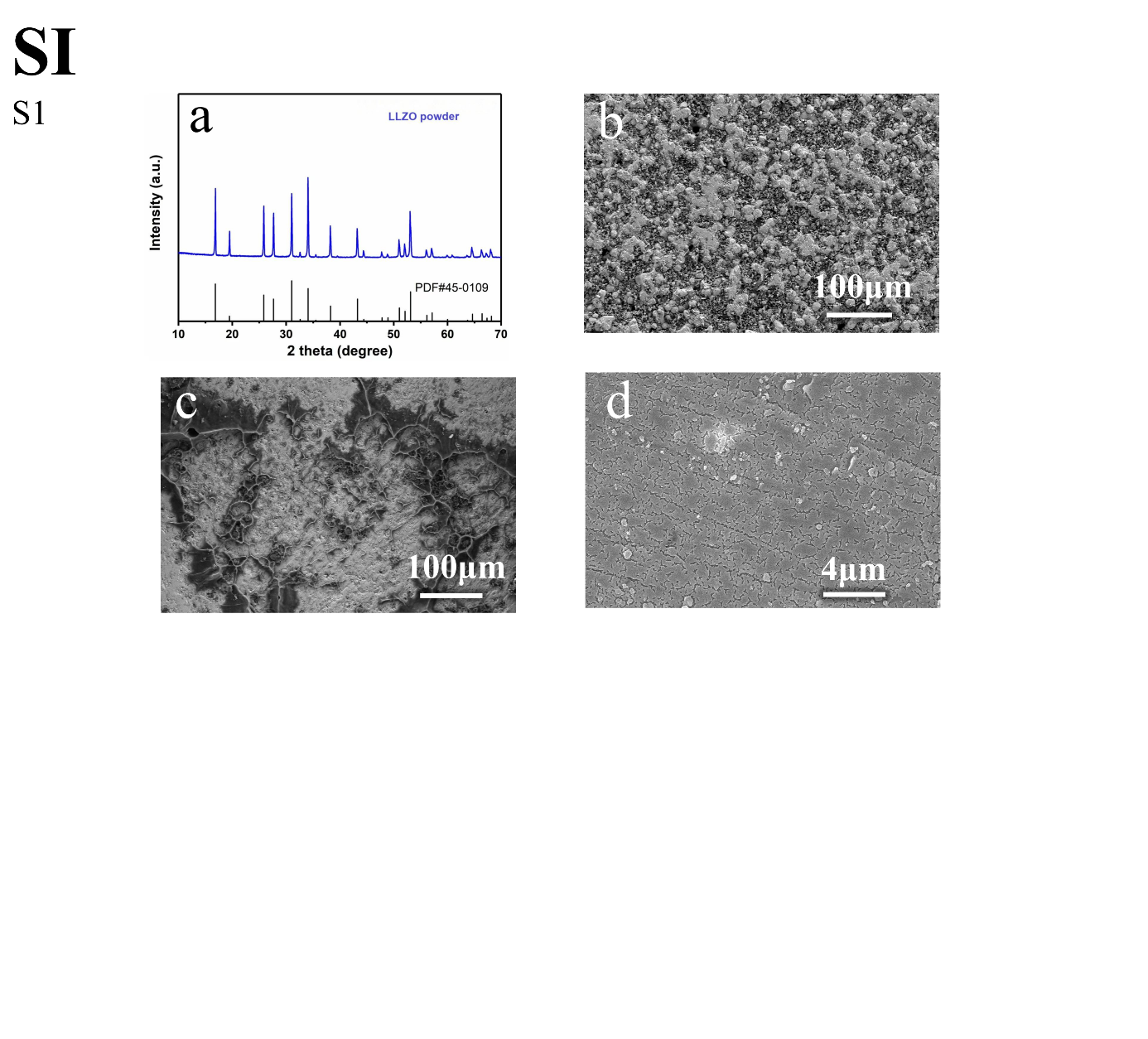


**Figure S2.** a) XRD pattern of the LLZO powder used in this study. SEM images of b) the original LLZO surface, c) the LLZO surface after ultrasonic treatment, and d) the LLZO surface after 200 hours of cycling.

**
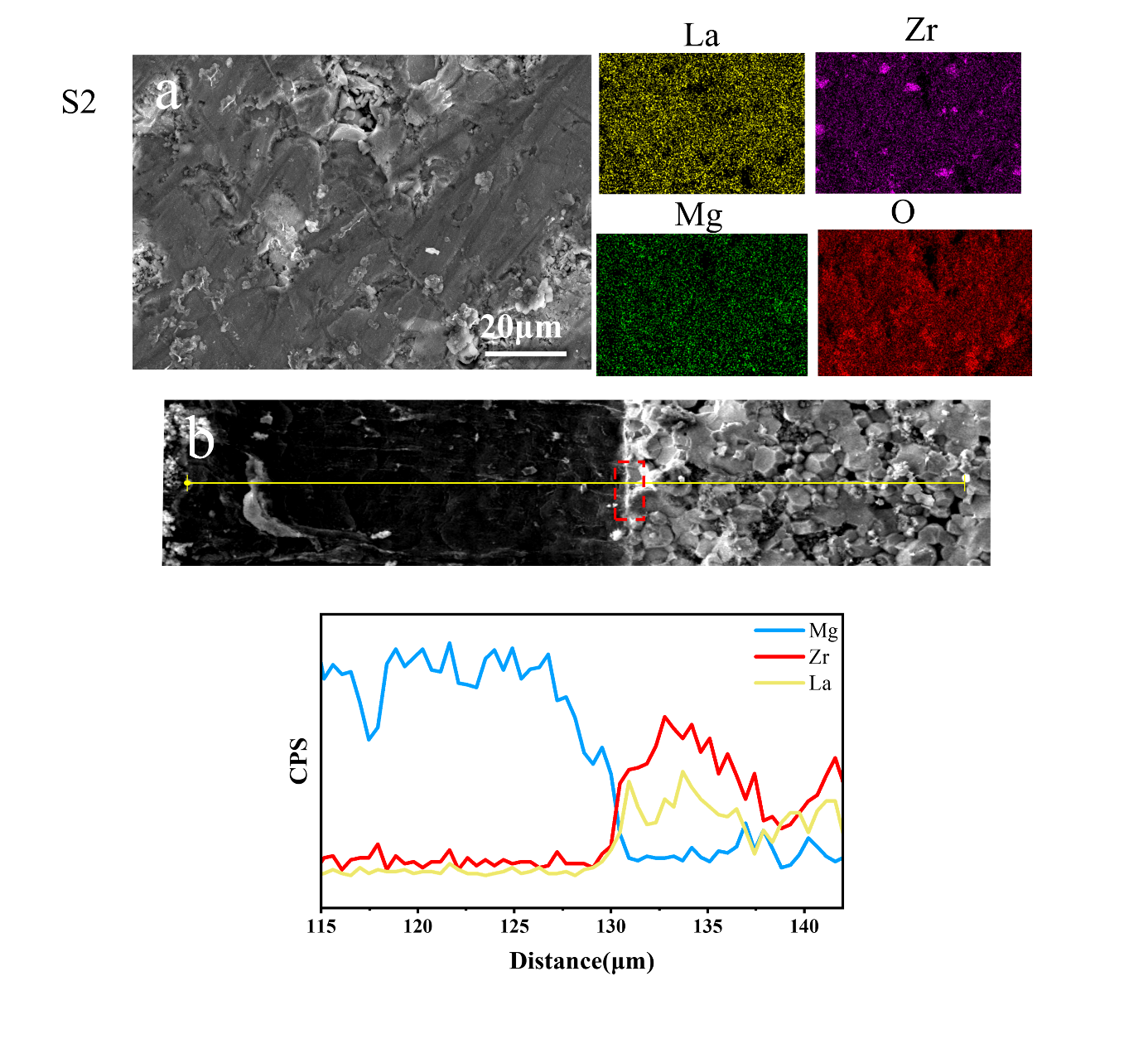
**

**Figure S3.** a) SEM images and EDS element mappings of the LLZO surface after ultrasonic treatment. b) EDS elemental line scan profile for UW-LiMg/LLZO.


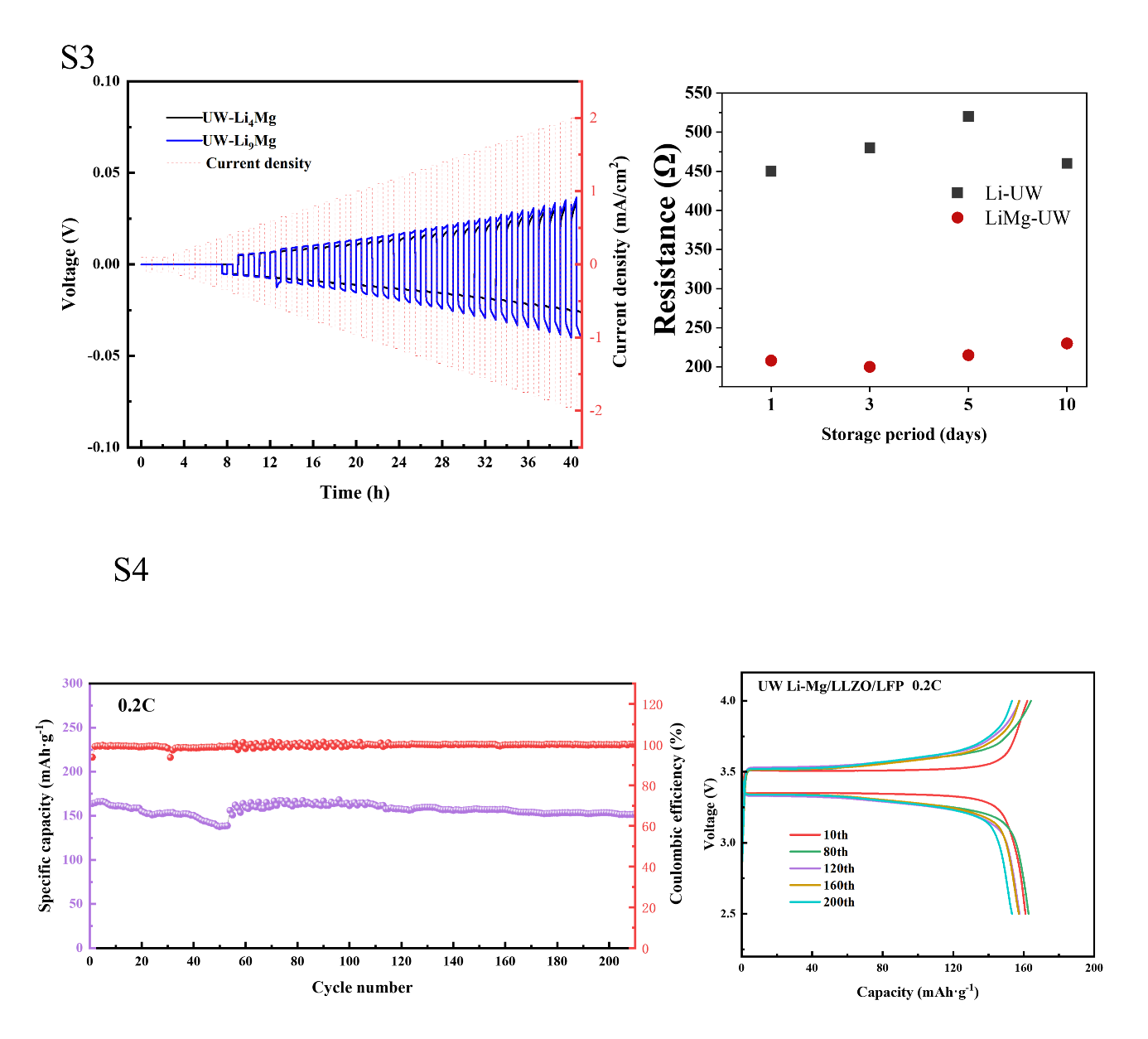


**Figure S4.** Cycling performance of the UW-LiMg/LLZO/LiFePO_4_ cell at 0.2 C and room temperature.


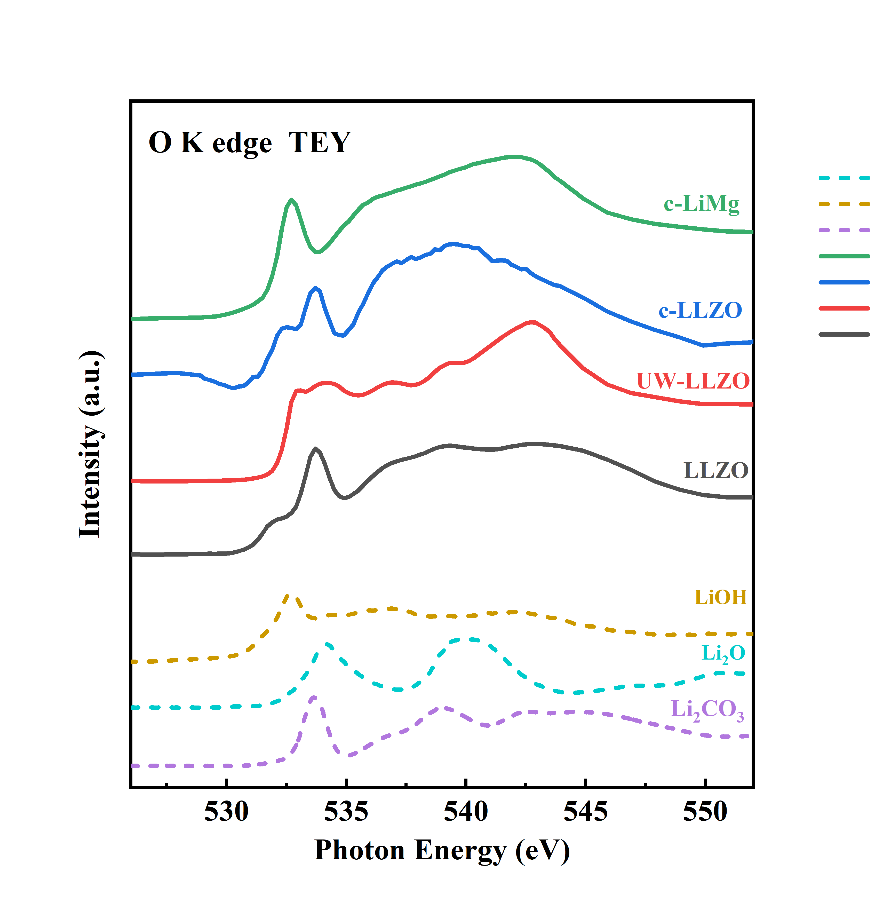


**Figure S5**. O K-edge XAS spectra in TEY mode of the surfaces of LLZO, UW-LLZO, c-LLZO, and c-LiMg. The detect depth is about 10 nm. LiOH, Li_2_O, and Li_2_CO_3_ are included as reference samples.

|  | interfacial resistance |
| --- | --- |
| This work | 65 Ω |
| Li-Na^[1]^ | 135 Ω |
| 3D Li^[2]^ | 750 Ω |
| UFS LLZO^[3]^ | 133 Ω |
| Ga_0.25_-LLZO^[4]^ | 180 Ω |
| BiCl_3_-LLZO- BiCl_3_^[5]^ | 110 Ω |
| Li-Sn^[6]^ | 187 Ω |

**Table S1.** Comparison of our interfacial resistance with results reported in other literature.

[1] X. Fu, T. Wang, W. Shen, M. Jiang, Y. Wang, Q. Dai, D. Wang, Z. Qiu, Y. Zhang, K. Deng, Q. Zeng, N. Zhao, X. Guo, Z. Liu, J. Liu, Z. Peng, A High-Performance Carbonate-Free Lithium/Garnet Interface Enabled by a Trace Amount of Sodium. *Adv. Mater.* **2020**, *32*, 2000575.

[2] S.-S. Chi, Y. Liu, N. Zhao, X. Guo, C.-W. Nan, L.-Z. Fan, Solid polymer electrolyte soft interface layer with 3D lithium anode for all-solid-state lithium batteries. *Energy Storage Mater.* **2019**, *17*, 309.

[3] H. Zhang, R. Dubey, M. Inniger, F. Okur, R. Wullich, A. Parrilli, D. T. Karabay, A. Neels, K. V. Kravchyk, M. V. Kovalenko, Ultrafast-sintered self-standing LLZO membranes for high energy density lithium-garnet solid-state batteries. *Cell Rep. Phys. Sci.* **2023**, *4*, 101473.

[4] S. Fu, P. Li, S. Yu, Y. Hu, Y. Liu, D. Chen, Y. Wei, Y. Li, Y. Chen, The effect of Ga doping on the microstructure and electrochemical properties of Li_7_La_3_Zr_2_O_12_ garnet-type solid electrolyte. *Solid State Ion.* **2025**, *420*, 116765.

[5] G. Zhao, C. Luo, Q. Hua, Li^+^ selective transport network-assisted high-performance of garnet-based solid electrolyte for Li metal batteries. *J. Mater. Chem. A* **2023**, *11*, 20174.

[6] J. Gao, C. Chen, Q. Dong, J. Dai, Y. Yao, T. Li, A. Rundlett, R. Wang, C. Wang, L. Hu, Stamping Flexible Li Alloy Anodes. *Adv. Mater.* **2021**, *33*, 5305.

|  | before cycle | after cycle |  |
| --- | --- | --- | --- |
| R_s_ (bulk impedance) | 86 Ω | 61 Ω |  |
| R_1_ (interfacial impedance) | 43 Ω | 115 Ω |  |
| R_2_ (charge transfer impedance) | 578 Ω | 664 Ω |  |

**Table S2.** The equivalent circuit fitting results of the EIS spectra in Figure 4b. The fitting results demonstrate that the interfacial impedance slightly increases from 43 Ω to 115 Ω after cycling, confirming the successful construction of a robust electrode/electrolyte interface. Moreover, the LiMg alloy effectively suppresses interfacial void formation, thereby stabilizing the interfacial resistance and significantly enhancing the cycling stability of the battery.
